# Orchestrating Physiological Homeostasis: The Spatial and Cellular Heterogeneity of Tryptophan Transport and Metabolism

**DOI:** 10.7150/ijbs.139760

**Published:** 2026-10-02

**Authors:** Xiaofeng Zhu, Yuling Zhang, Songlin Zhang, Sitong Chen, Yulong Yin, Xiongzhuo Tang

**Affiliations:** 1 Animal Nutritional Genome and Germplasm Innovation Research Center, College of Animal Science and Technology, Hunan Agricultural University, Changsha, Hunan 410128, China.; 2 Yuelushan Laboratory, Changsha, Hunan, 410128, China.; 3 Laboratory of Animal Nutritional Physiology and Metabolic Process, Key Laboratory of Agro-Ecological Processes in Subtropical Region, Institute of Subtropical Agriculture, Chinese Academy of Sciences, Changsha, Hunan, 410125, China.

**Keywords:** amino acid, enterochromaffin cell, tryptophan transport, hepatic first-pass

## Abstract

In addition to being incorporated into proteins, tryptophan (Trp) is increasingly recognized as a critical metabolic hub orchestrating systemic physiological homeostasis through interactions among the digestive, nervous and immune systems. This review systematically delineates the latest advancements in the molecular mechanisms governing the spatial and cellular heterogeneity of Trp transport. We summarize the sophisticated transmembrane machinery of Trp transport in intestinal epithelial cells, including angiotensin-converting enzyme 2-dependent broad neutral amino acid transporter 1 complex in absorptive enterocytes and L-type amino acid transporter 1/alanine, serine, cysteine transporter 2-mediated uptake in secretory enterochromaffin cells. Additionally, the albumin-binding dynamics of Trp in tryptophan 2,3-dioxygenase-mediated hepatic first-pass gating and its metabolic shunting to peripheral tissues are also discussed in this review. By bridging micro-level transport mechanisms with macro-level physiological regulation, this review provides a comprehensive framework for understanding the pleiotropic functions of Trp and identifies transporter-enzyme coupling as a potential target for therapeutic intervention in metabolic disorders, neurodegenerative diseases, and immune dysfunction.

## Introduction

Amino acids (AAs) are the building blocks of proteins and are essential for normal cellular growth and function 1. Tryptophan (Trp) is an essential amino acid (EAA) for maintaining nitrogen balance and facilitating protein synthesis 2. However, the versatile biological roles of Trp have been substantially revealed by recent metabolomic, immunological and neuroendocrinological research 3. The metabolic networks of Trp serve as a central hub that connects the neural, immune, and microbial systems in the host. Like in the gut, Trp-derived metabolites act together with other microbial generated bioactive compounds or particles including short chain fatty acids, secondary bile acids and extracellular vesicles that are essential for maintaining gut homeostasis and modulating immune functions 4-6.

These diverse functions are governed by specific membrane transporters and distinct metabolic networks. Intestinal Trp modulation exhibits substantial spatial and cellular heterogeneity due to the presence of specialized transporter systems within different intestinal epithelial compartments 7, 8. Absorptive enterocytes mediate Trp transport through dedicated membrane transporters, whereas secretory cells (such as enterochromaffin (EC) cells) convert intracellular Trp into 5-hydroxytryptamine (5-HT, serotonin) through tryptophan hydroxylase 1 (TPH1)-dependent metabolism 9, 10.

Within tissue and cellular microenvironments, Trp generates several vital bioactive compounds, predominantly through three core metabolic pathways: the kynurenine pathway (KP), 5-HT pathway, and gut microbiota-mediated indole pathway 11. These metabolites are central nodes for chemical signaling within the microbiota-gut-brain axis and play important roles in mediating energy metabolism, modulating inflammation and oxidative stress, inducing immune tolerance, and maintaining intestinal barrier homeostasis 9, 12.

Elucidating the transport and metabolic networks of Trp is essential for understanding metabolic diseases, neurodegenerative disorders, and immune dysregulation. Most current studies have focused on the host and microbiota mediated Trp metabolism. The mechanisms governing its transmembrane transport and tissue distribution remain incompletely defined. In this review, we first outline the major metabolic pathways of Trp and then focus on its transport processes in the host. As the first-pass effect describes how the liver extracts and metabolizes the absorbed Trp before it enters the systemic circulation 8, 10, 13, the route of Trp from intestinal absorption to hepatic portal circulation is discussed in this review. Finally, the distribution of Trp and uptake by peripheral tissues has also been included.

## Tryptophan Metabolic Pathways

Trp metabolism primarily involves two major systems: host cellular and gut microbiota metabolism (**Figure 1**). In host cells, Trp is mainly catabolized via two distinct pathways: the kynurenine and 5-HT pathways 4. Similar to other AAs, Trp metabolism is highly organ-specific and relies on distinct local enzymes 8. The liver is the central site of Trp metabolism, where the KP accounts for over 90% of total Trp degradation 14. The gastrointestinal tract and central nervous system (CNS) are secondary sites for Trp metabolism, in which the 5-HT pathway plays a pivotal role. More than 90% of the systemic 5-HT is synthesized in the gut, mainly by EC cells 15. The CNS contains less than 5% of total body 5-HT 16. In addition to host cellular metabolism, the gut microbiota can metabolize Trp. Therefore, unabsorbed Trp in the gut is primarily converted by the microbiota via the indole pathway 17.

### The Kynurenine Pathway

The KP is the principal pathway for Trp metabolism. The cascade of this pathway is initiated by the oxidative cleavage of the indole ring of Trp 18. This rate-limiting step is catalyzed either by tryptophan 2,3-dioxygenase (TDO, highly expressed in the liver, encoded by *TDO2*) or by indoleamine 2,3-dioxygenase 1 (IDO1, widely distributed in extrahepatic tissues and induced by inflammatory cytokines, such as interferon-γ [IFN-γ]) 18, 19. This reaction generates the unstable intermediate N-formylkynurenine, which is subsequently and rapidly hydrolyzed by arylformamidase (kynurenine formamidase) to yield L-kynurenine (L-Kyn), the central metabolite of the pathway 19. The downstream metabolism of L-Kyn primarily diverges into transamination, monooxygenation, and hydrolysis pathways 20. In the transamination pathway, L-Kyn is catalyzed by kynurenine aminotransferases to form kynurenic acid 20. In the monooxygenation branch, L-Kyn is first oxidized by kynurenine-3-monooxygenase to form 3-hydroxykynurenine, which is subsequently cleaved into 3-hydroxyanthranilic acid by kynureninase (KYNU) 12. In the direct hydrolytic pathway, L-Kyn is cleaved by KYNU to generate anthranilic acid 21.

### The Serotonin Pathway

Although the majority of Trp in the body is degraded via the KP, approximately 1-2% enters the 5-HT pathway 22. During the biosynthesis of 5-HT, Trp is hydroxylated to produce 5-hydroxytryptophan (5-HTP), a reaction catalyzed by the rate-limiting enzyme tryptophan hydroxylase (TPH) 23. Subsequently, 5-HTP is decarboxylated by aromatic L-amino acid decarboxylase (AADC) to generate the bioactive molecule, 5-HT 4. Furthermore, TPH exists as two distinct isoforms, TPH1 and tryptophan hydroxylase 2 (TPH2), which mediate 5-HT synthesis in peripheral tissues (primarily ECs) and the CNS, respectively 24-26. In addition to exerting direct receptor-mediated physiological functions, 5-HT is metabolized by monoamine oxidase (MAO) into 5-hydroxyindoleacetic acid, which is then excreted in urine 27.

### The Indole Pathway

Approximately 4-6% of unabsorbed dietary Trp reaches the colon, where it is metabolized into indole and its derivatives by the commensal gut microbiota 28. This process predominantly relies on the bacteria-expressed enzyme, tryptophanase, which catalyzes the cleavage of Trp into indole, pyruvate, and ammonia 29. In addition to indole, certain gut microbes use specialized enzymes to convert Trp into other active metabolites 30, 31. In one major branch, aromatic amino acid aminotransferase (ArAT) transforms Trp into the central intermediate indole-3-pyruvate (IPyA) 8, 32. From IPyA, several metabolic routes diverge. Decarboxylation by indolepyruvate decarboxylase (IpdC) yields indole-3-acetaldehyde, which is oxidized by indole-3-acetaldehyde dehydrogenase (IAAID-d) to produce indole-3-acetic acid (IAA) 4, 32. In another branch, IPyA is converted into indole-3-carboxaldehyde (IAld), primarily by *Lactobacillus* species, although the precise enzyme is unidentified 8, 30. The third pathway involves phenyllactate dehydrogenase (FldH), which reduces IPyA to indole-3-lactic acid (ILA). For many strains, metabolism ends at ILA. However, *Clostridium sporogenes* uses phenyllactate dehydratase (FldBC) to convert it into indole-3-acrylic acid (IA). Subsequently, acyl-CoA dehydrogenase (AcdA) reduces this IA into indole-3-propionic acid (IPA) 30, 32.

These three metabolic pathways determine the chemical fate of Trp in the host and their initiation and efficiency are dependent on the spatial availability of the substrate. This availability is governed by a highly orchestrated network of transmembrane transporters located in the intestinal epithelium, a primary site for nutrient absorption.

## Mechanisms of Intestinal Absorption, Transmembrane Transport, and Metabolism of Tryptophan

Most dietary Trp absorption occurs in the small intestine, especially in the jejunum and ileum 30. Intestinal epithelial cells (IECs) form the first cellular barrier between luminal Trp and the host circulation 33, 34. IECs comprise several distinct populations: the absorptive lineage, primarily consisting of enterocytes for nutrient uptake; the secretory lineage, which includes mucin-secreting goblet cells, antimicrobial peptide (AMP)-producing Paneth, and enteroendocrine cells (EECs); specialized functional or immune-related cells, such as chemosensory tuft and antigen-sampling microfold cells; and intestinal stem cells for epithelial renewal 35-37 (**Figure 2**). The fate of Trp differs between intestinal absorptive and secretory cells. To satisfy the systemic nutritional demands of the body, the absorptive lineage uses dedicated transporters to actively shuttle Trp across the membrane 38. In contrast, the secretory lineage, particularly EC cells, converts Trp into bioactive metabolites such as 5-HT, which contributes to intestinal motility and local immune and inflammatory regulation 39.

### Tryptophan Transport Pathways and Molecular Mechanisms in Intestinal Absorptive Cells

After ingestion by monogastric hosts, dietary proteins undergo rapid enzymatic cleavage by pepsin and pancreatic proteases. Subsequently, these massive complexes are stripped down into a readily absorbable pool consisting mostly of free amino acids (FAAs) and short oligopeptides, such as di- and tripeptides 40. Enterocytes, the dominant absorptive cell types among IECs, are critical sites for Trp uptake and its subsequent translocation into the systemic circulation. Efficient intestinal Trp uptake is important for growth and development 32. Notably, the efficiency of intestinal Trp transport not only determines its bioavailability, but also modulates its subsequent metabolic fate. It is the main physiological requirement for sustaining nitrogen balance and host homeostasis 41.

Under normal physiological conditions, Trp absorption is highly efficient; the majority is taken up by the small intestine, with only 4-6% reaching the colon for microbial degradation 28, 32. Trp absorption is a highly orchestrated energy-dependent physiological process regulated by the synergistic action of multiple carrier proteins, and involves both facilitated diffusion and active transport mechanisms. This process involves uptake at the brush border membrane (BBM) and efflux across the basolateral membrane (BLM), primarily relying on the coordinated expression and functional coupling of the solute carrier (SLC) family members 10 (**Figure 3**).

### Mechanism of Tryptophan Uptake via the Enterocyte Brush Border Membrane

The uptake of Trp across the enterocyte BBM is the main absorption step. Luminal free Trp is mainly transported across the BBM via the Na^+^-dependent transport, system B^0^ 42. The key transporter responsible for apical Trp uptake is broad neutral amino acid transporter 1 (B^0^AT1), encoded by the *SLC6A19* gene 43, 44.

B^0^AT1 is a broad-spectrum neutral AA symporter. It harnesses the Na^+^ electrochemical gradient established by the basolateral Na^+^/K^+^-ATPase to co-transport one Trp molecule and one Na^+^ ion into the cell 42, 45. The functional expression and localization of B^0^AT1 on the BBM do not occur independently; they are highly dependent on angiotensin-converting enzyme 2 (ACE2), which is a chaperone and accessory subunit to maintain its transport activity and membrane stability 23, 44. Because ACE2 is a key regulator of the renin-angiotensin system (RAS) 46, the RAS may participate in regulating Trp absorption via a non-classical pathway. The dependence of B^0^AT1 on ACE2 also makes this transport system sensitive to changes in ACE2 abundance. This becomes relevant during SARS-CoV-2 infection because ACE2 also serves as the viral entry receptor 23, 47. Viral binding and subsequent internalization are associated with reduced surface ACE2 expression. This disruption compromises B^0^AT1-ACE2 transport complex surface expression, potentially leading to impaired intestinal Trp absorption. Mechanistic studies indicate that localized Trp deficiency reduces epithelial antimicrobial peptide expression, promotes gut dysbiosis, and increases susceptibility to mucosal inflammation 48. This cascade pathology is a potential contributing explanation for the gastrointestinal symptoms frequently observed in patients with COVID-19 49, 50. Under normal dietary conditions, Trp also competes with other neutral amino acids for B^0^AT1-mediated uptake. These include other large neutral amino acids (LNAAs), particularly branched-chain amino acids (BCAAs, including leucine (Leu), isoleucine (Ile), and valine (Val)) and phenylalanine 38, 51, 52.

In addition to FAA transport, a portion of Trp is absorbed as Trp-containing di- or tri-peptides via peptide transporter 1 (PepT1) 53, 54. Encoded by *SLC15A1*, PepT1 is a high-capacity low-affinity transporter predominantly located in the BBM that mediates the transport of small peptides 55. PepT1 functions as a proton (H^+^)-coupled symporter. It co-transports one neutral or cationic di-peptide with one H^+^, whereas one anionic di-peptide is co-transported with two H^+^ ions 56, 57. The co-transported H^+^ is subsequently extruded by Na^+^/H^+^ exchanger 3 (NHE3, encoded by *SLC9A3*) on the BBM 58. Intracellular peptides are rapidly hydrolyzed by cytosolic peptidases into FAAs, which enter the intracellular AA pool 59. Furthermore, PepT1 expression is highly adaptable to dietary changes. Both high-protein diets and short-term fasting can upregulate PepT1 expression for peptide absorption 58. The absorption of peptide-bound Trp is a compensatory mechanism. If primary transporters, such as B^0^AT1, become saturated or pathologically impaired, the peptide-driven bypass provides an alternative absorptive route that can partially compensate for reduced amino acid uptake 40. This compensatory pathway helps preserve intestinal Trp uptake, thereby contributing to the maintenance of systemic Trp homeostasis and providing a mechanistic basis for improving precision nutrition. Therefore, future studies are needed to determine whether diets rich in Trp-containing dipeptides could exploit the PepT1 bypass to circumvent the ACE2/B^0^AT1 blockade and rescue acute AA starvation during viral enteritis.

The luminal substrate environment also affects apical Trp uptake. Dietary protein source and high-fat feeding can change the intestinal amino acid profile and the timing of substrate availability 60-63. These changes may modify the degree of substrate competition among LNAAs for B^0^AT1-mediated transport 38, 52, thereby influencing Trp absorption.

### Efflux and Uptake of Tryptophan at the Basolateral Membrane of Enterocytes

Following uptake by enterocytes via transporters on the BBM, free Trp is rarely involved in local synthesis or metabolism. Instead, it normally undergoes simple transcellular transit. It is directly exported across the BLM into the lamina propria, subsequently entering the capillaries and joining portal circulation 41. This efflux process primarily involves facilitated diffusion by T-type amino acid transporter 1 (TAT1, encoded by *SLC16A10*) and exchange transport by L-type amino acid transporter 2 (LAT2, encoded by *SLC7A8*) 40.

TAT1 is a specific passive uniporter for aromatic amino acids (AAAs) 64, 65. TAT1 channels intracellular Trp outward using only the transmembrane concentration gradient. This substrate-independent facilitated diffusion allows TAT1 to directly mediate net basolateral Trp efflux toward the portal circulation 64.

LAT2 typically forms a heterodimeric complex with the heavy chain, 4F2 cell-surface antigen heavy chain (4F2hc, alias for cluster of differentiation 98 (CD98), encoded by *SLC3A2*), via a disulfide bond to localize to the BLM and function properly 66. Unlike TAT1, LAT2 mainly functions as an amino acid exchanger rather than a simple efflux route. LAT2 exchanges intracellular and extracellular AAs in a 1:1 stoichiometric ratio 67. LAT2 mediates cellular uptake of neutral AAs, including glutamine (Gln), Trp, and Ile. This exchange directly couples the inward transport of these neutral AAs to the efflux of smaller, more polar AAs, such as glycine. Despite a minor capacity for unidirectional transport by LAT2, the actual efflux of AAAs, such as Trp and phenylalanine, is largely supported by TAT1-mediated facilitated diffusion 40.

### Transport Pathways and Molecular Mechanisms of Tryptophan in EECs

Although EECs account for only approximately 1% of IECs 68, EC cells play a pivotal role in Trp metabolism 69. Unlike enterocytes, which primarily mediate the transmembrane transport of Trp, EC cells, the largest endocrine cell population in the gut 70, specifically convert Trp into 5-HT, serving as a core node in the chemical signaling of the gut-brain axis 71.

### Uptake and Metabolism of Tryptophan in EC Cells

Trp required for 5-HT synthesis in EC cells is thought to be supplied largely from the basolateral circulation 72. In EC cells, Trp uptake is mediated primarily by L-type amino acid transporter 1 (LAT1, encoded by *SLC7A5*) 73, with alanine, serine, and cysteine transporter 2 (ASCT2, encoded by *SLC1A5*) indirectly supporting LAT1-dependent transport 74. Similar to LAT2, LAT1 associates with 4F2hc via a conserved disulfide linkage to form a heterodimeric complex that supports proper plasma membrane localization and transport function 75-77. LAT1 can utilize intracellular Gln as an exchange substrate, coupling Gln efflux to extracellular Trp uptake 75, 78. ASCT2 mediates Na^+^-dependent exchange of neutral AAs, including Gln, and helps maintain the intracellular exchange-substrate pool available to LAT1 79, 80. In EC cells, this arrangement may support LAT1-mediated Trp uptake and provide Trp for 5-HT synthesis (**Figure 4**).

This LAT1-ASCT2 coupling may stabilize Trp uptake when nutrient availability changes. ASCT2 maintains the intracellular Gln pool that LAT1 uses for amino acid exchange. This mechanism may help EC cells maintain 5-HT synthesis during fasting 81, 82.

Once Trp enters an EC cell, it can enter the 5-HT pathway. TPH1 first converts Trp to 5-HTP in the presence of tetrahydrobiopterin. AADC then converts 5-HTP to 5-HT 24, 82. Newly synthesized 5-HT is prone to degradation by MAO if left free in the cytoplasm. Therefore, it is rapidly sequestered into basolateral secretory granules by vesicular monoamine transporter 1 (encoded by *SLC18A1*) to form a high-concentration reserve that prevents intracellular oxidative degradation 69. The stored 5-HT can then be released when EC cells detect mechanical or chemical stimuli.

### Release of 5-HT from EC Cells

The release of 5-HT is primarily mediated by mechanical and chemical pathways 83, 84 (**Figure 5**). Mechanical forces associated with intestinal motility and luminal contents can stimulate mechanosensitive EC cells. Physical stretching directly induces the opening of Piezo2, a mechanosensitive ion channel in the EC cell membrane. This influx of nonselective cations (primarily Na^+^ and Ca^2+^) down their electrochemical gradients initiates cell membrane depolarization 84. When the membrane potential reaches the threshold for excitation, voltage-gated Na^+^ channels are activated, leading to Na^+^ influx and action potential generation 85. As action potentials propagate along the membrane, they rapidly activate voltage-gated calcium channels (VGCCs). This activation promotes extracellular Ca^2+^ influx and produces localized elevations in intracellular Ca^2+^ near secretory sites. Ultimately, this activates the soluble N-ethylmaleimide-sensitive factor attachment protein receptor (SNARE) complex, driving the exocytosis of 5-HT-containing dense-core vesicles and the release of 5-HT into the lamina propria 86, 87.

In the chemical-triggering pathway, the chemical microenvironment within the intestinal lumen, particularly nutrients and gut microbiota metabolites, is a crucial chemical stimulus for EC cells. Microbial metabolites, such as short-chain fatty acids (e.g., acetate, propionate, and butyrate), are sensed by G protein-coupled receptors in EC cells that possess an apical domain exposed to the intestinal lumen. These include olfactory receptor 78 (Olfr78, human homolog OR51E2) and the free fatty acid receptor 2, FFAR2 (also known as GPR43) 88. Olfr78 couples to Gs signaling. Activated Gs stimulates adenylyl cyclase and increases intracellular cyclic adenosine monophosphate (cAMP). Elevated cAMP activates protein kinase A (PKA), which can inhibit K^+^ channel activity and reduce outward K^+^ conductance. cAMP can also directly modulate hyperpolarization-activated cyclic nucleotide-gated (HCN) channels and enhance inward depolarizing cation currents. These two cAMP-dependent mechanisms favor membrane depolarization 89-91. In parallel, FFAR2 can couple to Gq and activate phospholipase C (PLC). PLC generates the second messenger inositol 1,4,5-trisphosphate (IP_3_), which mobilizes Ca^2+^ from the endoplasmic reticulum. The increase in cytosolic Ca^2+^ can activate depolarizing cation conductances and shift the membrane potential toward the threshold for action potential generation 92-94.

Locally, 5-HT released by EC cells activates 5-HT_3_ receptors on vagal and other primary afferent nerve fibers and thereby conveys sensory information along the gut-brain axis. 5-HT also acts on 5-HT_4_ and other serotonergic receptors within enteric neural circuits and contributes to peristaltic and secretory reflexes 85, 95. After completing local signal transmission, the released 5-HT is taken up by the serotonin transporter (SERT, encoded by *SLC6A4*), which is widely expressed in intestinal epithelial cells and subsequently intracellularly degraded by MAO 96. Systemically, 5-HT that is not cleared locally can enter the portal circulation 97. Circulating 5-HT is taken up by platelets via SERT and stored in platelets, whereas a much smaller free fraction remains in plasma 98. Platelets can release stored 5-HT upon activation 99. Free circulating gut-derived 5-HT can act as an endocrine signal in peripheral tissues. Gut-derived 5-HT has been implicated in bone formation and contributes to the regulation of glucose and lipid metabolism 81, 97, 100. These actions illustrate how Trp-derived 5-HT extends the physiological role of Trp beyond nutrient provision to systemic metabolic signaling. This is the fate of the Trp fraction used for intestinal 5-HT synthesis. Most absorbed Trp follows a different route and leaves the intestinal epithelium in unmetabolized form.

## Albumin Binding and Circulatory Transport of Tryptophan

After intestinal absorption, Trp enters the portal circulation. In portal blood, Trp already exists in free and albumin-bound forms. A large fraction binds reversibly to serum albumin, while a smaller fraction remains free in plasma 101. This equilibrium affects how much free Trp is available when portal blood reaches the liver (**Figure 6**). The same free-bound equilibrium continues after hepatic passage in the systemic circulation.

### Molecular Mechanism and Specificity of Tryptophan Binding to Albumin

Under physiological conditions, approximately 80-95% of Trp in plasma is non-covalently bound to serum albumin, leaving only 5-20% in the free form 102. Trp primarily binds to Sudlow site II (the indole-benzodiazepine site) on the albumin molecule, which is located within the hydrophobic pocket of subdomain IIIA 103. This binding is saturable and governed by the law of mass action, maintaining a dynamic dissociation-association equilibrium. The albumin-bound pool acts as a circulating reservoir and replenishes free Trp as tissues remove it from blood 104, 105.

Albumin binding also limits Trp loss from the circulation. The albumin-Trp complex crosses the glomerular filtration barrier poorly because of its large molecular size. Albumin binding therefore reduces renal Trp loss and limits the amount of Trp that is immediately available to enter catabolic pathways in the liver and other tissues. These effects help maintain a relatively stable circulating Trp pool 101, 106. The binding is reversible 104. As tissues take up free Trp, albumin releases part of its bound Trp and replenishes the free pool. Free Trp is the main form available for tissue uptake. It can be taken up by peripheral tissues such as the liver and skeletal muscle through amino acid transporters. Similarly, free Trp must cross the blood-brain barrier (BBB) through specific amino acid transport systems to enter the CNS 107, 108. As free Trp is taken up by tissues, albumin-bound Trp can contribute to replenishment of the free pool through reversible dissociation and thereby help maintain circulating Trp availability.

### Factors That Alter Albumin-Tryptophan Binding

The distribution of Trp between free and albumin-bound pools is influenced by plasma non-esterified fatty acid (NEFA) concentrations. Trp binds mainly to Sudlow site II on albumin. The FA3-FA4 region overlaps with this site and binds fatty acids. Albumin also has several high-affinity sites for long-chain fatty acids 101, 109, 110. NEFAs can compete with Trp for sites on albumin 111. Fasting, stress, and prolonged exercise can increase plasma NEFA levels. High-fat feeding can also raise plasma NEFA levels under some conditions 111-113. Higher NEFA levels can displace Trp from albumin and increase the free Trp fraction. Disease-associated glycation and oxidation can alter human serum albumin (HSA) structure and ligand binding at Sudlow site II. For instance, glycation changes the conformation of this site and alters its affinity for ligands in patients with diabetes 114, 115. Oxidative modification also changes albumin structure and impair Site II binding for some ligands 116. The Sudlow site II acts as the major binding site for Trp and structural changes at this site can alter the equilibrium between albumin-bound Trp and free Trp, thus changing the circulating free Trp pool 101. Subsequently, the changed free Trp pool in portal blood reaches the liver, where part of the absorbed Trp is removed during the first pass.

### Hepatic First-Pass Effect and Cell-Specific Metabolic Shunting

Portal blood reaches the liver before the systemic circulation. During this first pass, the liver extracts and metabolizes part of the absorbed Trp. The remainder enters the systemic circulation 8, 10, 13 (**Figure 7**). Direct quantitative data on hepatic first-pass extraction of Trp in humans remain limited. In sheep, the liver extracts approximately 78% of Trp from the portal-drained viscera, leaving only 22% for the systemic circulation 117.

This first-pass extraction differs from the liver's cumulative contribution to whole-body Trp catabolism. The liver is the primary organ for Trp catabolism (accounting for approximately 90% of the total degradation) and serves as a metabolic hub. Through hepatic uptake and TDO-mediated catabolism, the liver regulates how much Trp is degraded locally and how much remains available to the systemic circulation 10, 102.

Hepatic Trp metabolism exhibits substantial cellular heterogeneity 118. Under homeostatic conditions, Trp crosses the hepatic sinusoidal endothelium into the Space of Disse 119 and is subsequently taken up via the TAT1 transporter located at the sinusoidal membrane of hepatocytes 120. The intracellular metabolic flux of Trp through the kynurenine pathway is largely regulated by the rate-limiting enzyme TDO 121. At baseline physiological Trp concentrations, TDO-mediated catabolism is restrained, which helps preserve Trp availability in the systemic circulation. Conversely, Trp availability rises after ingestion of Trp-rich protein meals 122. When Trp availability rises, increased substrate supply can enhance TDO-mediated Trp catabolism in hepatocytes.

Beyond this substrate-dependent hepatocyte response, an additional IDO1-mediated route of hepatic Trp catabolism can be induced during inflammation. The portal circulation continuously exposes the liver to gut-derived pathogen-associated molecular patterns (PAMPs), including lipopolysaccharide (LPS), and periportal Kupffer cells help restrain excessive responses to these signals 123. During inflammation, IFN-γ can induce IDO1 in Kupffer cells and dendritic cells, where IDO1 promotes local Trp catabolism, suppresses T-cell responses, and may contribute to hepatic immune tolerance 124, 125. Thus, IDO1 in hepatic immune cells provides an inflammation-sensitive complement to TDO-mediated control of hepatic Trp metabolism. These findings suggest that inflammatory signaling can shift hepatic Trp metabolism toward local IDO1-dependent catabolism, potentially reducing the amount of Trp that reaches the systemic circulation. This may constitute a gut-liver regulatory link through which inflammatory stress favors local immunoregulatory Trp catabolism over peripheral Trp availability.

### Tissue-Targeted Transport and Metabolism of Tryptophan Following the Hepatic First-Pass Effect

Although the liver is the primary site of Trp catabolism and TDO is predominantly expressed in the liver, a fraction of absorbed Trp remains unmetabolized after hepatic passage and enters the systemic circulation 10, 126. This fraction, here referred to as “post-hepatic Trp,” undergoes further metabolism in peripheral tissues. Although this post-hepatic fraction is smaller than the amount of Trp metabolized by the liver, it remains physiologically important because it supports protein synthesis and serves as a substrate for tissue-specific pathways involved in neuroendocrine and immune regulation 8.

### Nervous System

Within the nervous system, the CNS is a critical target organ for post-hepatic Trp metabolism. Trp cannot freely diffuse across the BBB. Instead, its entry into the brain parenchyma is mediated mainly by LAT1 expression in brain capillary endothelial cells 108, 127. At the BBB, Trp competes with other LNAAs for LAT1-mediated transport 128, 129. Brain 5-HT synthesis depends not only on dietary Trp intake but also on the plasma Trp/LNAA ratio, which influences Trp availability for LAT1-mediated transport across the BBB 130.

High-protein diets typically provide substantially more competing LNAAs, particularly BCAAs, relative to Trp. This can lower the plasma Trp/LNAA ratio and increase competition for LAT1 at the BBB. As a result, less Trp may become available for brain uptake 122, 130. Lower brain Trp availability can reduce brain 5-HT synthesis 131. Conversely, carbohydrate-rich, low-protein meals can stimulate insulin secretion and promote the uptake of competing LNAAs, particularly BCAAs, by peripheral tissues. This can increase the plasma Trp/LNAA ratio and facilitate Trp uptake into the brain, increasing its availability for 5-HT synthesis 132, 133. The same competitive transport mechanism also becomes evident in liver disease. In patients with liver cirrhosis and hepatic encephalopathy, plasma BCAA levels are decreased relative to levels of aromatic amino acids (AAAs), which further alters the profile of circulating LNAAs 134, 135. As BCAAs and AAAs belong to the group of LNAAs that compete for LAT1 at the BBB, this imbalance reduces competition from BCAAs and favors brain uptake of AAAs, including Trp 135, 136. Additionally, BCAA-enriched infusion also reduces Trp transport and 5-HT levels in cerebrospinal fluid 136. Recent targeted metabolomics further demonstrate the systemic dysregulation of Trp and serotonin metabolism in patients with cirrhosis and hepatic encephalopathy 137. These findings indicate that pathological changes in the plasma LNAA profile can alter Trp delivery to the brain and consequently modify central 5-HT metabolism. Thus, the Trp/LNAA-LAT1 mechanism described above for dietary regulation also has clinical relevance in liver dysfunction and hepatic encephalopathy. At the BBB, competition for LAT1 controls how much circulating Trp enters the brain. In tumors and immune tissues, local Trp consumption becomes another major determinant of Trp availability.

### Immune System and Tumor Microenvironment

In the tumor microenvironment (TME), malignant cells can increase Trp uptake through upregulated amino acid transporters 138. Tumor and immune cells that express IDO1 or TDO can also enhance local Trp catabolism, reduce Trp availability, and increase the production of kynurenine-pathway metabolites 139. IFN-γ can further induce IDO1 in antigen-presenting and tumor cells 140. Reduced Trp availability can limit effector T-cell proliferation. Trp depletion also activates the general control nonderepressible 2 (GCN2)-eukaryotic initiation factor 2α (eIF2α)-activating transcription factor 4 (ATF4) amino-acid stress response and induces GCN2-dependent upregulation of LAT1 74, 141. In the kynurenine-rich TME, increased LAT1 expression enhances kynurenine uptake, which promotes aryl hydrocarbon receptor (AHR) signaling and favors regulatory T-cell (Treg) differentiation 141. Strikingly, despite a strong mechanistic rationale, clinical development of IDO1 inhibitors has yielded disappointing results, a failure that may be partially attributable to compensatory upregulation of upstream transporters such as LAT1, which sustains kynurenine delivery to the tumor microenvironment. For instance, in the Phase 3 ECHO-301/KEYNOTE-252 trial, the addition of epacadostat to pembrolizumab failed to improve progression-free or overall survival compared with pembrolizumab monotherapy 142. This outcome indicates that selective IDO1 inhibition alone may be insufficient to dismantle the broader Trp-Kyn-AHR immunoregulatory network. Clinical epacadostat treatment induces adaptive metabolic changes within the TME 143. These findings support therapeutic strategies that target multiple nodes of the Trp-Kyn pathway. Dual IDO1/TDO inhibition can counter compensatory TDO activity, whereas pharmacological blockade of LAT1 can limit kynurenine uptake and restore antitumor T-cell function in preclinical tumor models 144, 145.

The expression of LAT1 also changes during non-neoplastic intestinal inflammation, but its role is different from that in tumors. In active ulcerative colitis, LAT1 expression is increased in regenerating colonic mucosa, particularly in the lower crypt region 146. Recent experimental evidence further shows that LAT1 supports epithelial recovery from intestinal inflammation, as LAT1 deficiency delays tissue repair and impairs colonic crypt regeneration 147. Therefore, increased LAT1 expression may help intestinal epithelial cells meet their amino acid demand.

### Peripheral Organ Utilization: Muscle Anabolic Metabolism, Renal Reabsorption, and Placental Transfer

Peripheral organs use Trp in different ways. Skeletal muscle contributes to amino acid utilization, the kidney limits urinary Trp loss, and reproductive tissues transfer amino acids to the fetus or milk.

### Skeletal Muscle Anabolism

Skeletal muscle is the largest peripheral amino-acid reservoir and contributes potentially to systemic Trp modulation through protein turnover and tissue Trp/Kyn metabolism 10, 148. Insulin can modulate skeletal-muscle amino-acid handling by increasing the inward transport of selected amino acids and promoting muscle protein anabolism 149. At the sarcolemma, LAT1 function is closely coupled to sodium-coupled neutral amino acid transporter 2 (SNAT2, encoded by *SLC38A2*) in skeletal-muscle amino-acid transport 150. In skeletal muscle, insulin can also increase LAT1 expression through a mechanistic target of rapamycin complex 1 (mTORC1)-dependent mechanism in skeletal muscle cells 151. SNAT2 complements this regulation by mediating Na^+^-coupled Gln uptake and increasing intracellular Gln availability. Gln can then serve as an exchange substrate for LAT1-mediated uptake of extracellular LNAAs, including Trp 152. This mechanism may therefore contribute to intracellular Trp availability in skeletal muscle and to the amino-acid pool available for protein synthesis. In the condition of metabolic dysfunction, however, the regulation of the LAT1/SNAT2 transport network may be altered. Insulin-resistant myotubes accumulate extracellular BCAAs without changing the abundance of LAT1, indicating that amino acid disorders cannot be explained by reduced LAT1 level alone 153. Additionally, aging also blunts the increased expression of SNAT2 after resistance exercise and EAA ingestion 154. These findings imply that insulin resistance and aging are capable of altering the LAT1/SNAT2-dependent amino acid transport, thus further affecting Trp delivery to skeletal muscle and contributing to anabolic resistance.

### Renal Reabsorption

Although the intestinal tract is the initial site of absorption, renal reabsorption also contributes to plasma Trp homeostasis by limiting urinary Trp loss 10. At the renal proximal tubule BBM, B^0^AT1 plays a major role in Trp reabsorption 155. Furthermore, TAT1 and LAT2 on the BLM are responsible for the efflux of reabsorbed Trp back into systemic circulation 156.

### Reproductive System Remodeling in Placental Barrier and Mammary Tissue

During gestation and lactation, tissue-specific AA transport systems adapt to changing nutrient demands, including the increased demand for Trp. In the placenta, System L transporters within the syncytiotrophoblast form a major route for maternal-fetal Trp transfer. In human term placenta, LAT1 is predominantly localized to the maternal-facing microvillous membrane (MVM), whereas LAT2 is detected at both the MVM and the fetal-facing basal membrane (BM) 157. At the MVM, System L transporters, particularly LAT1, mediate Trp uptake from maternal blood through amino-acid exchange. Placental Trp transport is a critical rate-limiting step that governs placental Trp availability and metabolism, and fetal Trp supply 158. After entry into the syncytiotrophoblast, Trp must cross the BM to reach the fetal circulation 158.

During lactation, mammary AA transport also adapts to support milk protein synthesis. Prolactin increases SNAT2 expression, and SNAT2 protein abundance can rise by more than 10-fold during early lactation in rat mammary tissue 159. SNAT2 transports neutral AAs such as Gln, which can expand the intracellular pool of exchange substrates available to System L. Prolactin also increases LAT1 expression and plasma-membrane localization in mammary epithelial cells and enhances LAT1-dependent AA uptake 160. Coordinated SNAT2 and LAT1 activity may also support mammary Trp uptake during lactation and contribute to the amino-acid supply required for milk protein synthesis.

## Conclusions and Perspectives

The spatial and cellular heterogeneity of tryptophan transport and metabolism account for numerous physiological and pathological functions in the host. Trp modulation within host cells or organs involves multiple sequential steps, including intestinal absorption, portal circulation, reversible albumin binding, hepatic first-pass effect, and ultimate distribution to peripheral tissues. These processes collectively determine the Trp availability for protein synthesis or its downstream metabolic processes.

The allocation of Trp levels and the influence of transporter on its metabolic fate within individual tissues have gained increasing attention. Circulating Trp levels alone only offer a preliminary estimate of its tissue-specific bioavailability. Rather, local Trp levels are determined by the integrated actions of transporter activity, substrate competition, albumin binding, and intracellular metabolism. Recently, genetically encoded Trp sensors such as green ratiometric indicator have been shown to permit quantitative measurement of Trp dynamics in living biological systems 2, 161. Further development of these tools may enable direct comparisons of extracellular, intracellular, and tissue-specific Trp pools, thereby discriminating whether altered Trp levels are determined by transport kinetics or by metabolic consumption.

Trp transport in absorptive enterocytes is relatively well defined, particularly the roles of B^0^AT1 at the brush-border membrane and TAT1/LAT2 at the basolateral membrane. In contrast, the transport machinery utilized by EC cells is less completely established. The proposed LAT1-ASCT2 interaction provides a plausible mechanism for Trp uptake, while much of the evidence is inferred from transporter biochemistry and lacks experimental validation in EC cells. Moreover, far less is known regarding Trp acquisition by other intestinal epithelial populations, including Paneth cells, goblet cells, tuft cells and intestinal stem cells. Defining the transporter repertoire of these cell types in combination with measurement of cell-specific Trp levels will be essential to elucidate how Trp is distributed and functions among the heterogeneous intestinal epithelial compartment.

The interaction between intestinal transport and microbial metabolism of Trp also requires further investigation. Human studies indicate the association between dietary Trp intake and circulating microbial metabolites such as indole-3-propionic acid 17. Experimental studies further reveal a close relationship between intestinal amino-acid transport and the gut microbiota. In *Chd8^+/-^*mice, changes in microbial composition are associated with altered expression of intestinal amino-acid transporter and circulating amino-acid levels. Likewise, microbiota manipulations also modify intestinal amino-acid transport 40, 162. However, the molecular signals responsible for these effects remain unclear. Whether specific microbial species or their metabolites directly regulate the expression, membrane localization, or transport activity of Trp transporters such as the B^0^AT1-ACE2 complex remains unexplored. Dissecting these mechanisms would establish the crosstalk between microbial metabolism of Trp and its transport process in intestinal epithelial cells.

Notably, the mechanism of how Trp is extracted in the process of hepatic first-pass in humans remains incompletely characterized. Current understanding is mainly based on studies focused on hepatocyte uptake and TDO-dependent Trp metabolism under normal condition as well as inducible IDO1-dependent Trp catabolism upon inflammatory responses in the liver. Additionally, the mechanisms of Trp-specific transport in peripheral tissues also lack investigation. For instance, the proposed models of Trp modulation in skeletal muscle and mammary tissue are established by neutral amino acid transport rather than direct transport of Trp. Future studies should quantify the proportion of Trp extracted during the first hepatic passage in humans and investigate how tissue-specific Trp distribution is determined by dietary Trp availability, inflammatory stimulation, and diseased conditions.

Finally, Trp transporters have been considered as potential therapeutic targets in recent years. For instance, Trp depletion increases GCN2-dependent LAT1 expression and Kyn uptake and enhances AHR-dependent Treg cell differentiation 141. Pharmacological inhibition of LAT1 by compound nanvuranlat (JPH203) has been shown to restrict nutrient acquisition by tumor cells in a clinical trial 163. Whether LAT1 inhibition modifies the Kyn-AHR immunoregulatory axis in patients remains to be established. Because LAT1 requires its association with 4F2hc for stable plasma-membrane localization and transport 75, disrupting the LAT1-4F2hc interaction may provide an alternative clinical strategy for limiting LAT1 activity. In addition, the gut microbiota-derived metabolites are also capable of modulating the activity of Trp transporters for disease treatment. For instance, indole-3-propionic acid has been shown to inhibit the activity of SLC36 to suppress tumor growth 164. Engineered *Clostridium sporogenes* enhances indole-3-propionic acid production to treat postmenopausal osteoporosis 165. Thus, targeting specific microbial metabolites may provide a new therapeutic strategy for disease treatment or control.

## Figures and Tables

**Figure 1:**
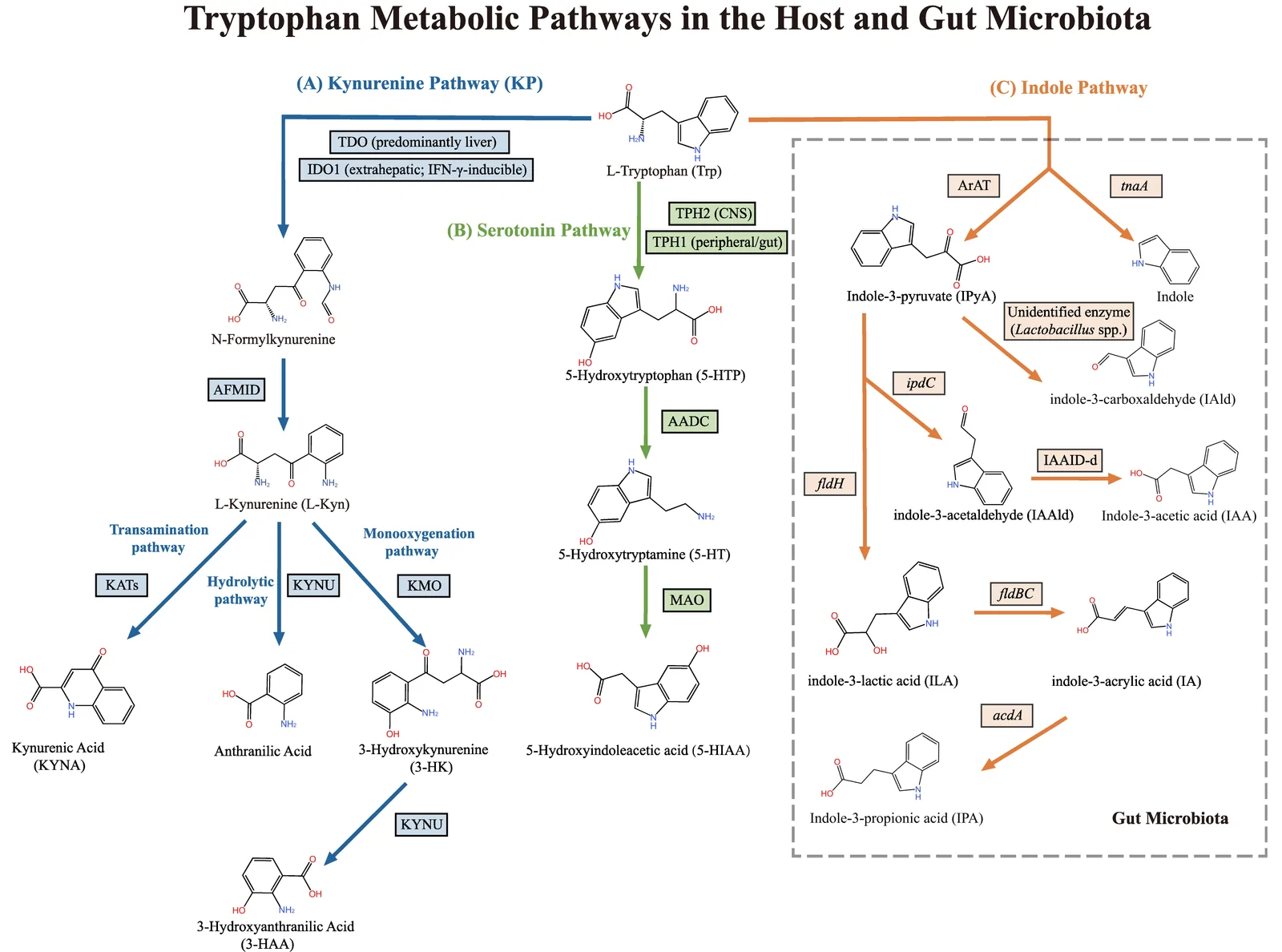
** The Metabolic Pathways of Tryptophan in the Host and Gut Microbiota.** Host cells and gut microbes metabolize Trp through three major pathways. (A) The kynurenine pathway is the principal route of host Trp catabolism. TDO acts mainly in the liver, whereas IDO1 is widely expressed in extrahepatic tissues and can be induced by inflammatory cytokines such as IFN-γ. Both enzymes catalyze the first step from Trp to N-formylkynurenine. AFMID then converts N-formylkynurenine to L-Kyn. KATs convert L-Kyn to KYNA, KYNU converts L-Kyn to anthranilic acid, and KMO converts L-Kyn to 3-HK. KYNU further converts 3-HK to 3-HAA. (B) Approximately 1-2% of Trp enters the serotonin pathway. TPH1 acts mainly in peripheral tissues, especially gut enterochromaffin cells, whereas TPH2 functions in the CNS. Both enzymes convert Trp to 5-HTP. AADC converts 5-HTP to 5-HT, and MAO metabolizes 5-HT to 5-HIAA. (C) About 4-6% of dietary Trp reaches the colon. Gut microbes convert this fraction to indole and related metabolites. TnaA converts Trp to indole, whereas ArAT converts Trp to IPyA. IpdC converts IPyA to IAAld, and IAAID-d converts IAAld to IAA. A separate branch forms IAld, mainly in *Lactobacillus* spp. The responsible enzyme remains unidentified. FldH converts IPyA to ILA, FldBC converts ILA to IA, and AcdA converts IA to IPA. Abbreviations: Trp, tryptophan; IFN-γ, interferon-γ; TDO, tryptophan 2,3-dioxygenase; IDO1, indoleamine 2,3-dioxygenase 1; AFMID, arylformamidase; L-Kyn, L-kynurenine; KATs, kynurenine aminotransferases; KYNA, kynurenic acid; KMO, kynurenine 3-monooxygenase; 3-HK, 3-hydroxykynurenine; KYNU, kynureninase; 3-HAA, 3-hydroxyanthranilic acid; TPH1, tryptophan hydroxylase 1; TPH2, tryptophan hydroxylase 2; CNS, central nervous system; 5-HTP, 5-hydroxytryptophan; AADC, aromatic L-amino acid decarboxylase; 5-HT, 5-hydroxytryptamine; MAO, monoamine oxidase; 5-HIAA, 5-hydroxyindoleacetic acid; *tnaA*, gene encoding tryptophanase; ArAT, aromatic amino acid aminotransferase; IPyA, indole-3-pyruvate; *ipdC*, gene encoding indolepyruvate decarboxylase; IAAld, indole-3-acetaldehyde; IAAID-d, indole-3-acetaldehyde dehydrogenase; IAA, indole-3-acetic acid; IAld, indole-3-carboxaldehyde; *fldH*, gene encoding phenyllactate dehydrogenase; ILA, indole-3-lactic acid; *fldBC*, gene encoding phenyllactate dehydratase; IA, indole-3-acrylic acid; *acdA*, gene encoding acyl-CoA dehydrogenase; IPA, indole-3-propionic acid.

**Figure 2:**
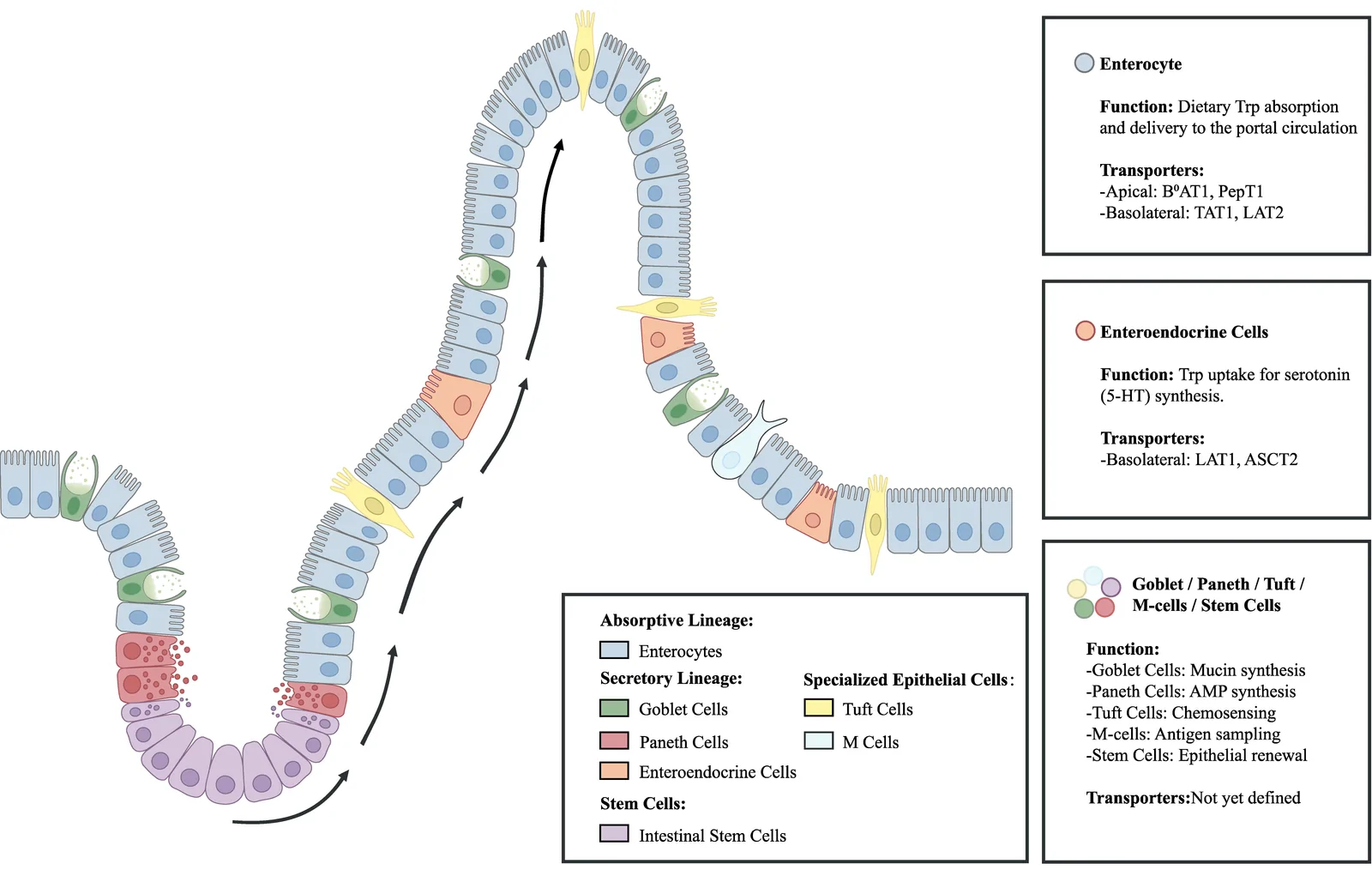
** Spatial Distribution, Cellular Heterogeneity, and Transporter Annotation of Intestinal Epithelial Cells in the Jejunum and Ileum.** The intestinal epithelium has distinct lineages. Enterocytes, the primary absorptive site, mediate the directional transcellular shuttling of luminal Trp. This directional transport is powered by specific apical uptake (B^0^AT1 and PepT1) and is functionally coupled with basolateral efflux (TAT1 and LAT2). The secretory lineages include goblet, Paneth, and enteroendocrine cells (EECs). Among EECs, enterochromaffin cells (EC) rely on basolateral transporters (LAT1 and ASCT2) for Trp uptake to synthesize bioactive signaling molecules, such as serotonin. Cells with special functions (tuft and microfold cells) coordinate localized mucosal immunity. Concurrently, the intestinal stem cells at the crypt base drive epithelial renewal. However, the Trp transporters in the goblet, Paneth, tuft, microfold, and stem cells remain unclear. Abbreviations: AMP, antimicrobial peptide; ASCT2, alanine, serine, cysteine transporter 2; B0AT1, broad neutral amino acid transporter 1; EC, enterochromaffin; EECs, enteroendocrine cells; LAT1/2, L-type amino acid transporter 1/2; M cells, microfold cells; PepT1, peptide transporter 1; TAT1, T-type amino acid transporter 1; Trp, tryptophan; 5-HT, 5-hydroxytryptamine.

**Figure 3:**
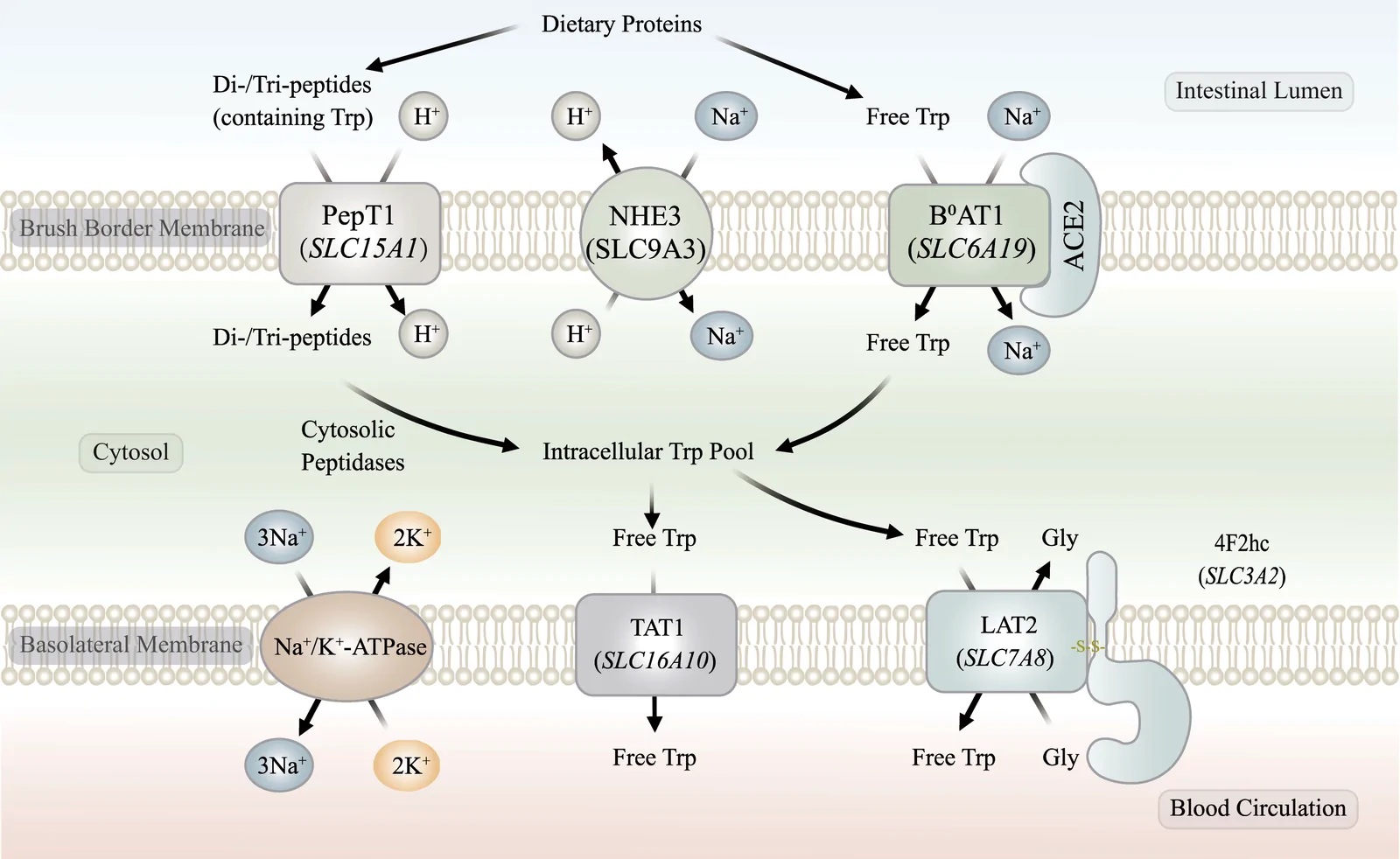
** Molecular Mechanisms of Tryptophan Transmembrane Transport in Absorptive Enterocytes.** At the brush border membrane, free luminal Trp is primarily cotransported with Na^+^ by the B^0^AT1 transporter (encoded by *SLC6A19*). B^0^AT1 depends on ACE2 for proper membrane localization and transport function. Di- and tripeptides (Trp-containing) are internalized via the H^+^-coupled peptide transporter, PepT1 (encoded by *SLC15A1*), and hydrolyzed by cytosolic peptidases into free Trp. NHE3 exports H^+^ in exchange for luminal Na^+^ and helps maintain the proton gradient required for PepT1 activity. The basolateral Na^+^/K^+^-ATPase maintains the Na^+^ gradient that supports apical transport. At the basolateral membrane, the efflux of Trp into portal circulation is primarily facilitated by the uniporter, TAT1 (encoded by *SLC16A10*). The antiporter, LAT2 (encoded by *SLC7A8*), which is covalently linked to the heavy chain, 4F2hc (encoded by *SLC3A2*), primarily functions as an exchanger to maintain the intracellular AA pool. Abbreviations: B^0^AT1, broad neutral amino acid transporter 1; ACE2, angiotensin-converting enzyme 2; PepT1, peptide transporter 1; NHE3, Na^+^/H^+^ exchanger 3; AA, amino acid; TAT1, T-type amino acid transporter 1; LAT2, L-type amino acid transporter 2; 4F2hc, 4F2 cell-surface antigen heavy chain; Gly, glycine; Trp, tryptophan.

**Figure 4:**
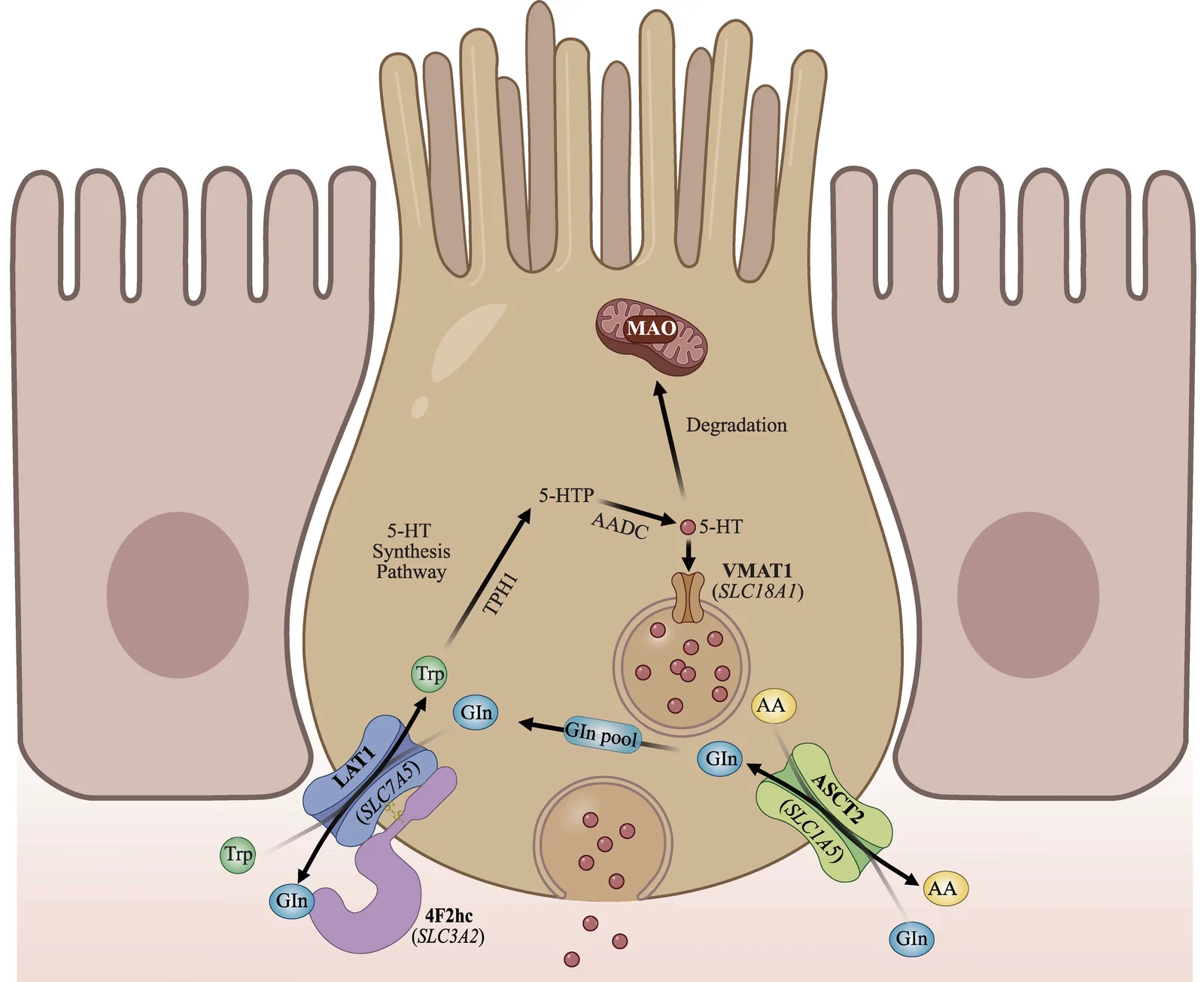
** Metabolic Coupling of Tryptophan Uptake and Serotonin Biosynthesis in Enterochromaffin Cells.** Trp for serotonin synthesis in ECs is supplied largely from the basolateral circulation. LAT1 (encoded by *SLC7A5*) forms a complex with 4F2hc (encoded by *SLC3A2*) and exchanges intracellular glutamine (Gln) for extracellular Trp. ASCT2 (encoded by *SLC1A5*) supports this process by maintaining the intracellular Gln pool available for LAT1 exchange. TPH1 converts intracellular Trp to 5-hydroxytryptophan (5-HTP), and AADC converts 5-HTP to serotonin (5-HT). Free cytosolic 5-HT is susceptible to MAO-mediated degradation. VMAT1 (encoded by *SLC18A1*) transports newly synthesized 5-HT into secretory granules for storage and subsequent release. Abbreviations: AADC, aromatic L-amino acid decarboxylase; ASCT2, alanine, serine, cysteine transporter 2; 4F2hc, 4F2 cell-surface antigen heavy chain; Gln, glutamine; LAT1, L-type amino acid transporter 1; MAO, monoamine oxidase; TPH1, tryptophan hydroxylase 1; Trp, tryptophan; 5-HT, 5-hydroxytryptamine; 5-HTP, 5-hydroxytryptophan; AA, amino acid; ECs, enterochromaffin cells; VMAT1, vesicular monoamine transporter 1.

**Figure 5:**
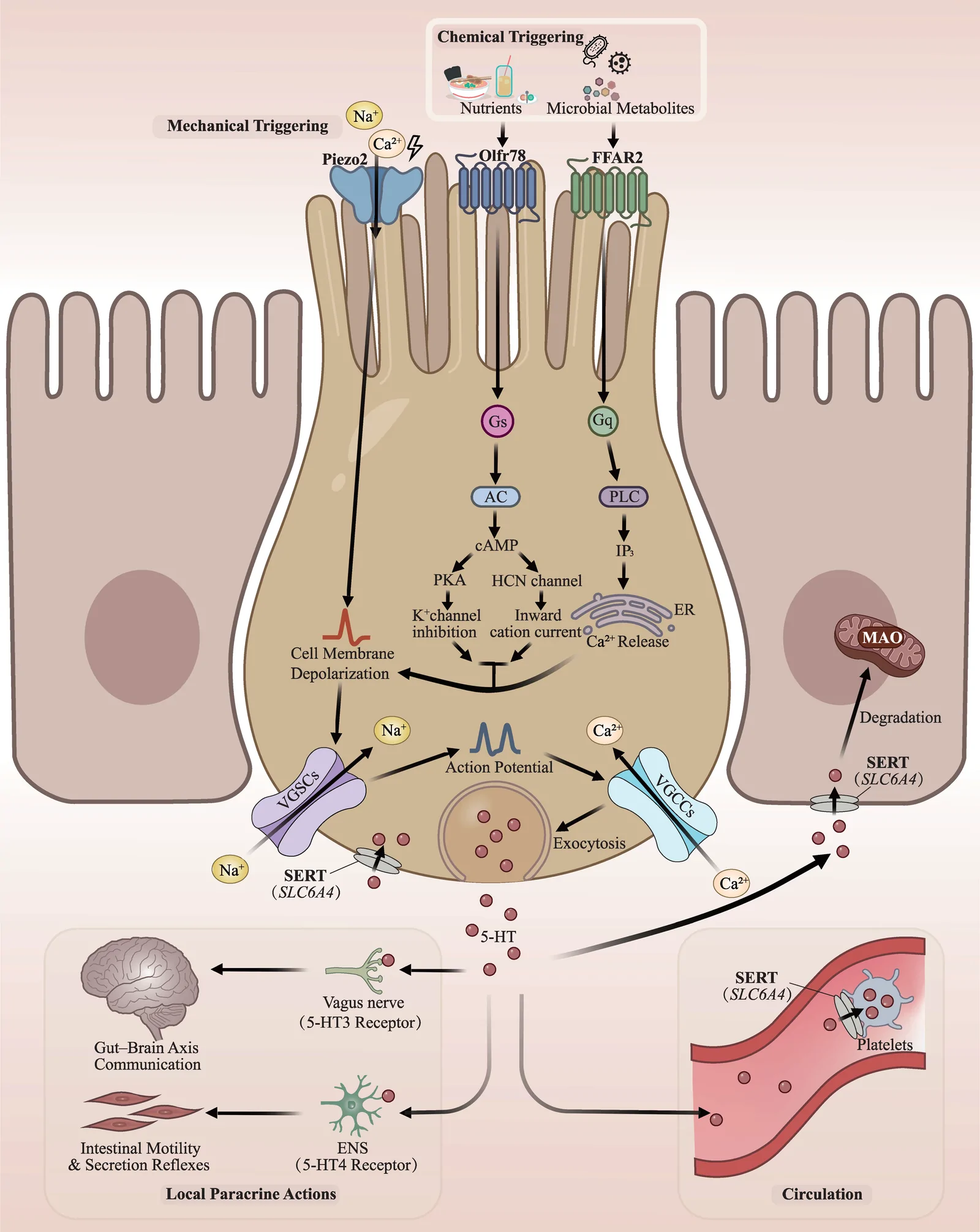
** Mechanosensory and Chemosensory Control of Serotonin Release from Enterochromaffin Cells and Its Downstream Actions.** Mechanical and chemical signals trigger 5-HT release from ECs. Mechanical triggering: Intestinal stretch activates Piezo2 and allows Na^+^ and Ca^2+^ to enter the cell. The resulting membrane depolarization activates voltage-gated sodium channels (VGSCs) and generates action potentials. Voltage-gated calcium channels (VGCCs) then open and increase Ca^2+^ influx. The rise in intracellular Ca^2+^ triggers SNARE-mediated exocytosis of 5-HT-containing vesicles. Chemical triggering: Luminal nutrients and microbial metabolites provide chemical stimuli to EC cells. Olfr78 couples to Gs. Gs activates adenylyl cyclase (AC) and increases cAMP. cAMP activates PKA, which reduces K^+^ conductance, and also enhances inward depolarizing currents through HCN channels. FFAR2 couples to Gq and activates PLC. PLC generates IP_3_, which releases Ca^2+^ from the endoplasmic reticulum and promotes membrane depolarization. Released 5-HT activates 5-HT_3_ receptors on vagal afferents and 5-HT_4_ and other serotonergic receptors in enteric neural circuits. These local signals support gut-brain communication and intestinal motility and secretion. Intestinal epithelial cells take up extracellular 5-HT through SERT, followed by intracellular degradation by MAO. 5-HT that escapes local clearance can enter the circulation, where platelets take it up through SERT and store it. Abbreviations: AC, adenylyl cyclase; cAMP, cyclic adenosine monophosphate; ECs, enterochromaffin cells; ER, endoplasmic reticulum; FFAR2, free fatty acid receptor 2; Gs/Gq, G-protein subtypes; HCN, hyperpolarization-activated cyclic nucleotide-gated; IP_3_, inositol 1,4,5-trisphosphate; MAO, monoamine oxidase; Olfr78, olfactory receptor 78; PKA, protein kinase A; PLC, phospholipase C; SERT, serotonin transporter; SNARE, soluble N-ethylmaleimide-sensitive factor attachment protein receptor; VGCCs, voltage-gated calcium channels; VGSCs, voltage-gated sodium channels; ENS, enteric nervous system; 5-HT, 5-hydroxytryptamine.

**Figure 6:**
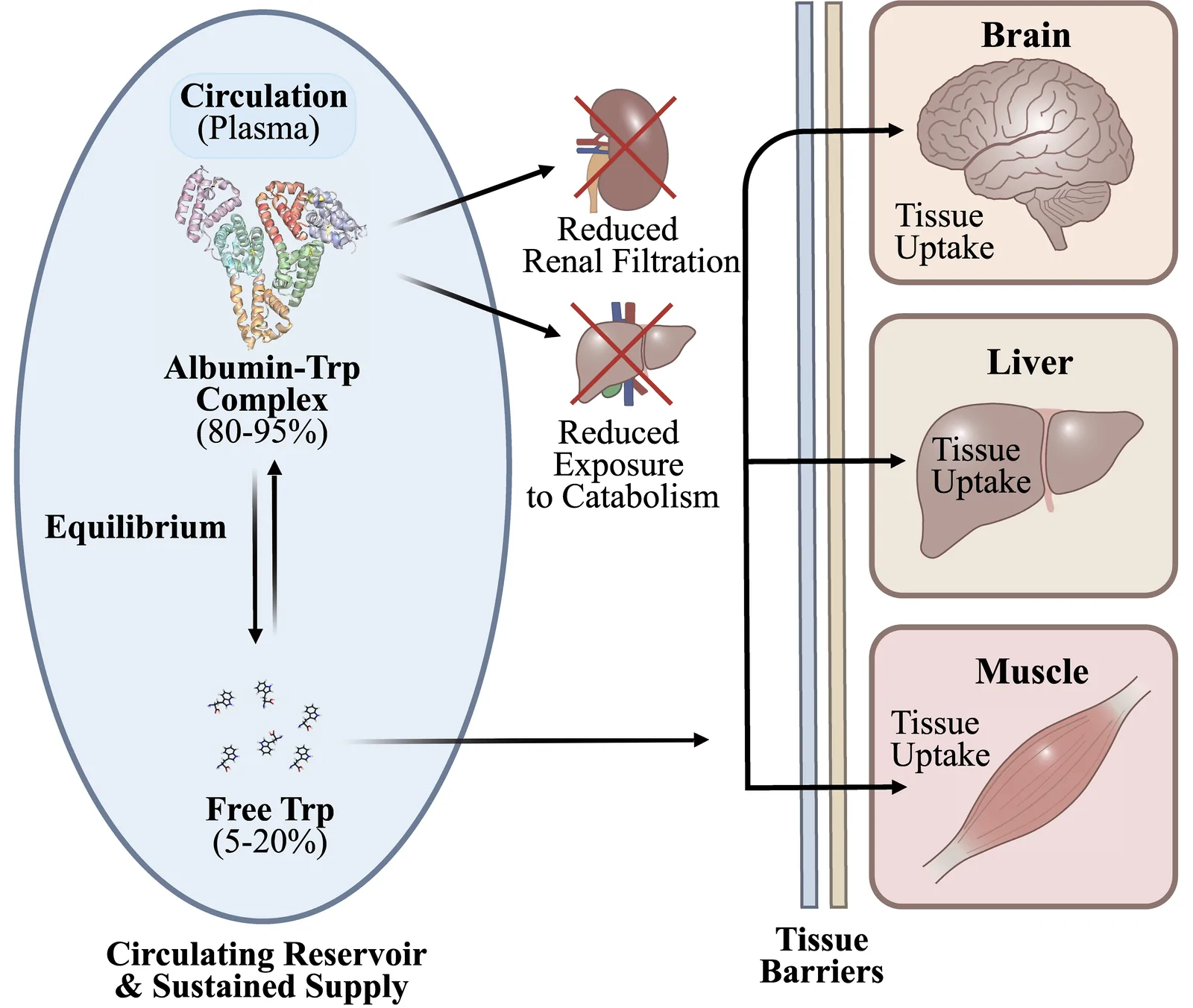
** Circulatory Transport Forms and Albumin-Binding Dynamics of Tryptophan.** Plasma Trp exists as albumin-bound and free fractions that remain in reversible equilibrium. About 80-95% is bound to serum albumin, mainly at Sudlow site II, whereas 5-20% remains free. Albumin-bound Trp forms a circulating reservoir for the free pool. Albumin binding reduces renal Trp loss and limits exposure to catabolic pathways. Free Trp is taken up by peripheral tissues such as the liver and skeletal muscle and can enter the brain after transport across the BBB. Abbreviations: BBB, blood-brain barrier; Trp, tryptophan.

**Figure 7:**
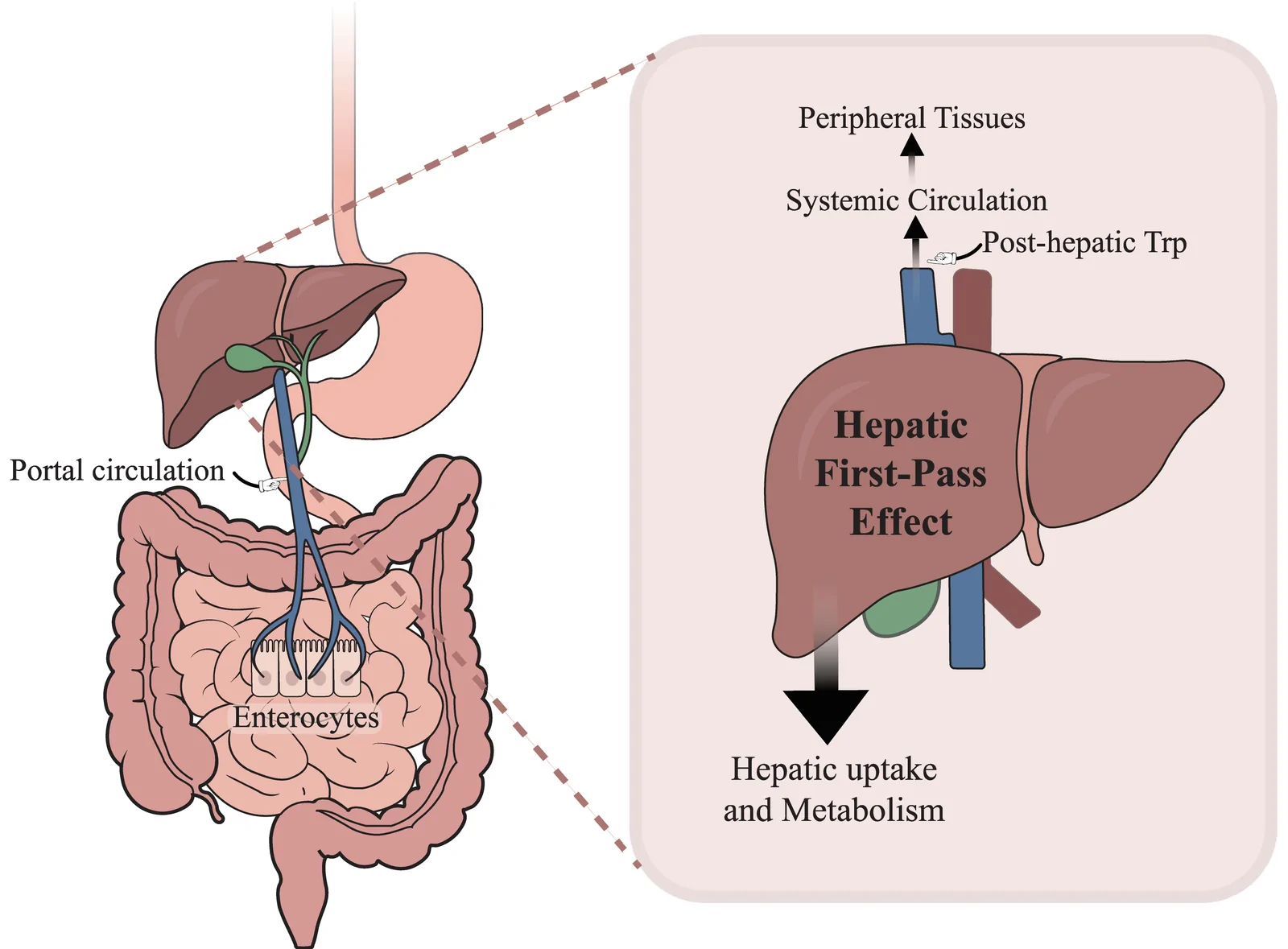
** Hepatic First-Pass Effect and Post-Hepatic Distribution of Tryptophan.** After intestinal absorption, Trp enters the portal circulation and reaches the liver before the systemic circulation. The liver takes up and metabolizes part of the absorbed Trp during this first pass. The remaining Trp leaves the liver and enters the systemic circulation for delivery to peripheral tissues. The extent of first-pass Trp extraction in humans remains poorly defined. The liver also accounts for a major share of whole-body Trp catabolism, but this cumulative contribution is distinct from first-pass extraction. Abbreviation: Trp, tryptophan.

## Abbreviations

3-HAA: 3-hydroxyanthranilic acid
3-HK: 3-hydroxykynurenine
4F2hc: 4F2 cell-surface antigen heavy chain
5-HIAA: 5-hydroxyindoleacetic acid
5-HT: 5-hydroxytryptamine
5-HTP: 5-hydroxytryptophan
AAAs: aromatic amino acids
AADC: aromatic L-amino acid decarboxylase
AAs: amino acids
*acdA*: gene encoding acyl-CoA dehydrogenase
ACE2: angiotensin-converting enzyme 2
AFMID: arylformamidase
AHR: aryl hydrocarbon receptor
AMP: antimicrobial peptide
ArAT: aromatic amino acid aminotransferase
ASCT2: alanine, serine, cysteine transporter 2
ATF4: activating transcription factor 4
B^0^AT1: broad neutral amino acid transporter 1
BBB: blood-brain barrier
BBM: brush border membrane
BCAAs: branched-chain amino acids
BLM: basolateral membrane
BM: basal membrane
cAMP: cyclic adenosine monophosphate
CD98: cluster of differentiation 98 (alias for 4F2hc)
CNS: central nervous system
CSF: cerebrospinal fluid
EAA: essential amino acid
EC: enterochromaffin
EECs: enteroendocrine cells
eIF2α: eukaryotic initiation factor 2α
FAAs: free amino acids
FFAR2: free fatty acid receptor 2
*fldBC*: gene encoding phenyllactate dehydratase
*fldH*: gene encoding phenyllactate dehydrogenase
GCN2: general control nonderepressible 2
Gln: glutamine
HCN: hyperpolarization-activated cyclic nucleotide-gated
HSA: human serum albumin
*ipdC*: gene encoding indolepyruvate decarboxylase
IA: indole-3-acrylic acid
IAA: indole-3-acetic acid
IAAld: indole-3-acetaldehyde
IAAID-d: indole-3-acetaldehyde dehydrogenase
IAld: indole-3-carboxaldehyde
IDO1: indoleamine 2,3-dioxygenase 1
IECs: intestinal epithelial cells
IFN-γ: interferon-γ
ILA: indole-3-lactic acid
Ile: isoleucine
IP_3_: inositol 1,4,5-trisphosphate
IPA: indole-3-propionic acid
ipdC: indolepyruvate decarboxylase
IPyA: indole-3-pyruvate
KATs: kynurenine aminotransferases
KMO: kynurenine 3-monooxygenase
KP: kynurenine pathway
KYNA: kynurenic acid
KYNU: kynureninase
LAT1: L-type amino acid transporter 1
LAT2: L-type amino acid transporter 2
Leu: leucine
L-Kyn: L-kynurenine
LNAAs: large neutral amino acids
LPS: lipopolysaccharide
mTORC1: mechanistic target of rapamycin complex 1
MAO: monoamine oxidase
MVM: microvillous membrane
NEFAs: non-esterified fatty acids
NHE3: Na^+^/H^+^ exchanger 3
Olfr78: olfactory receptor 78
PAMPs: pathogen-associated molecular patterns
PepT1: peptide transporter 1
PLC: phospholipase C
PKA: protein kinase A
RAS: renin-angiotensin system
SERT: serotonin transporter
SLC: solute carrier
SNARE: soluble N-ethylmaleimide-sensitive factor attachment protein receptor
SNAT2: sodium-coupled neutral amino acid transporter 2
*tnaA*: gene encoding tryptophanase
TAT1: T-type amino acid transporter 1
TDO: tryptophan 2,3-dioxygenase
TME: tumor microenvironment
TPH1: tryptophan hydroxylase 1
TPH2: tryptophan hydroxylase 2
TPH: tryptophan hydroxylase
Tregs: regulatory T cells
Trp: tryptophan
Val: valine
VGCCs: voltage-gated calcium channels
